# Interference competition as a key determinant for spatial distribution of mangrove crabs

**DOI:** 10.1186/s12898-018-0164-1

**Published:** 2018-02-15

**Authors:** Stefano Cannicci, Marco Fusi, Filippo Cimó, Farid Dahdouh-Guebas, Sara Fratini

**Affiliations:** 1 The Swire Institute of Marine Science and the School of Biological Sciences, The University of Hong Kong, Pokfulam Road, Hong Kong, Hong Kong SAR; 2 Department of Biology, University of Florence, via Madonna del Piano 6, 50019 Sesto Fiorentino, Italy; 3 Biological and Environmental Sciences and Engineering Division (BESE), King Abdullah University of Science and Technology (KAUST), Thuwal, 23955-6900 Saudi Arabia; 4 Laboratory of Systems Ecology and Resource Management, Université Libre de Bruxelles-ULB, Av. F.D. Roosevelt 50, CPi 264/1, 1050 Brussels, Belgium; 5 Laboratory of Plant Biology and Nature Management, Mangrove Management Group, Vrije Universiteit Brussel-VUB, Pleinlaan 2, 1050 Brussels, Belgium; 6 Mangrove Specialist Group, IUCN Species Survival Commission, 28 rue Mauverney, 1196 Gland, Switzerland

**Keywords:** Sesarmid crabs, Aggressive behaviour, Distribution patterns, Indo-Pacific mangroves, Environmental factors

## Abstract

**Background:**

The spatial distribution of mangrove crabs has been commonly associated with tree zonation and abiotic factors such as ground temperature and soil granulometry. Conversely, no studies were designed to investigate the role of competition for resources and predation in shaping crab distribution in mangroves, despite these biotic factors are recognised as key determinants for spatial patterns observed in the communities colonising rocky and sandy intertidal habitats.We studied floral and faunal assemblages in two zones of a Sri Lankan mangrove, a man-made upper intertidal level and a natural eulittoral, mid-shore one. Leaf choice experiments were designed to study both feeding rate and intra and inter-specific interactions for food of sesarmid crabs in the two habitats in order to better understand crab spatial distribution.

**Results:**

The two intertidal belts differed in terms of floral composition and crab species abundance. The eulittoral zone was strongly dominated by *Neosarmatium smithi*, while within the elevated littoral fringe four sesarmids (*N. smithi*, *N. asiaticum*, *N. malabaricum* and *Muradium tetragonum*) were more evenly distributed. At both levels, all sesarmids showed to collect significantly more *Bruguiera* spp. and *Rhizophora apiculata* leaves than *Excoecaria agallocha* ones. There was no temporal segregation in feeding activity among the four species, resulting in a high interference competition for leaves. Regardless of the habitat, *N. smithi* was always successful in winning inter-specific fights.

**Conclusions:**

Our results showed that the elevated littoral fringe was more crowded with crabs, but was less favourable in terms of food availability and environmental conditions. The dominance of *N. smithi* in gathering mangrove leaves suggests that this species may segregate the other sesarmids into less favourable habitats. The present data strongly suggest for the first time that interference competition for food can contribute to shape mangrove crab spatial distribution.

## Background

Indo-Pacific mangrove forests host abundant populations of molluscs and crabs, both critically important taxa in energetics and food web [1–3]. Respect to molluscs, brachuyuran crabs are also extremely diverse in terms of genera and species [3]. In the Indian subcontinent, for instance, 149 species, belonging to 75 genera were found living in mangroves [4], while over a hundred species are known to colonise mangroves of the peninsular Malaysia [5]. This rather diverse crab fauna exhibits some degrees of spatial segregation that is often related to intertidal zones, similarly to what can be observed on less spatially complex intertidal habitats [6, 7]. This view was developed after the classical studies by Macnae [6], Sasekumar [8] and Hartnoll [9], who divided the mangrove forests in major zones differing in terms of tidal level, inundation time, floral composition and soil texture and described crab assemblages typical of each zone. Since those papers, a considerable amount of literature confirmed the distribution patterns of the Indo-Pacific mangrove crabs [10–13] gathering a huge amount of descriptive data on brachyuran zonation in mangroves. However, very few studies paralleled these qualitative analyses with sound quantitative or experimental approach aimed at testing which environmental factors can affect the spatial distribution of the different species. In addition, most of these few studies dealt only with the fiddler crabs, genus *Uca* sensu lato [14], whose distribution patterns are thought to be controlled by their differential resistance to high temperatures [11, 15–17] and by their morphological specialisation for deposit-feeding, resulting in a substratum-dependent spatial segregation [10, 18, 19].

On the other hand, factors affecting sesarmid crab distribution were rarely addressed, even if these crabs are by far the most abundant and biodiverse macrobenthic taxon within the mangroves and they proved to be very important species in terms of litter consumption [e.g. 20–22], ecosystem engineering [3, 23–27] and propagule predation [28–31].

Observations on species distribution and spatial segregation along the forest, coupled with data on their feeding preferences, suggested a strong correlation between the presence of some major litter consumers, such as the crabs of the Indo-Pacific genus *Neosarmatium*, and the presence of their preferred trees [6, 7]. In this view, the patterns of tree distribution present in various part of the Indo-Pacific region was hypothesised to be the principal drivers of sesarmid crab distribution. Cannicci et al. [3] and Dahdouh-Guebas et al. [32] suggested that crab distribution is the result of a complex interaction between different biotic and abiotic environmental factors, while Paula et al. [33] stressed the importance of the effects of stratified larval recruitment and competence in their distribution. Therefore, no specific hypotheses were formulated on the influence of factors such as competition for resources and predation, known to be key determinant of zonation in other intertidal environments [34–36].

Competition for resources (e.g. food, mates and space) is a major regulatory factor of population dynamics and structure. Competition among foragers can be exploitative, when it is an indirect competition by reducing available food items [37]. Conversely, it is referred to as interference competition when foragers directly interact to access food [37, 38]. Interference competition has been commonly observed in nature either at intra- and inter-specific level and generally is reported as asymmetric, with superior individuals gaining more resources and segregating inferior individuals to less favourable sites [39]. Therefore, the distribution and structure of local populations can be theoretically affected by both exploitative and interference competition as discussed in Fretwell and Lucas [40]. The consequences of interference competition remain however poorly understood [see 39].

Within this framework, sesarmid crab populations are ideal systems to test hypotheses on the effect of inter-specific competition on their distribution within mangroves. In mangroves, in fact, many closely related species live in sympatry, share the same activity windows, have access to the same limited food resource (i.e. leaf litter) and indirectly and directly compete, at intra- and inter-specific level, for food [21, 41]. Fratini et al. [21], for example, studied the feeding behaviour of mangrove crabs in a high competitive environment, and found that one species adopted the strategy of stealing leaves from foraging individuals belonging to smaller species. However, the authors never investigated the effect of such competition for food on species distributions [21].

Herein we report the results of a series of measures of environmental parameters, field observations and feeding experiments addressing the role of interference competition in the spatial distribution of litter-feeding crabs of the genera *Neosarmatium* and *Muradium* (e.g. the most relevant species feeding on mangrove fallen leaves in the Indo-Pacific mangroves) within a Sri Lankan mangrove forest. This study took advantage of intrinsic natural environmental differences in terms of abiotic factors and community composition characterising different zones of the study site. Although resource competition is historically well-recognised as an important factor shaping intertidal community organization [42], this is the first experimental attempt aimed at assessing the role of inter-specific interference competition for food in mangrove crab distribution.

## Methods

### Study area and experimental sites

**Fig. 1:**
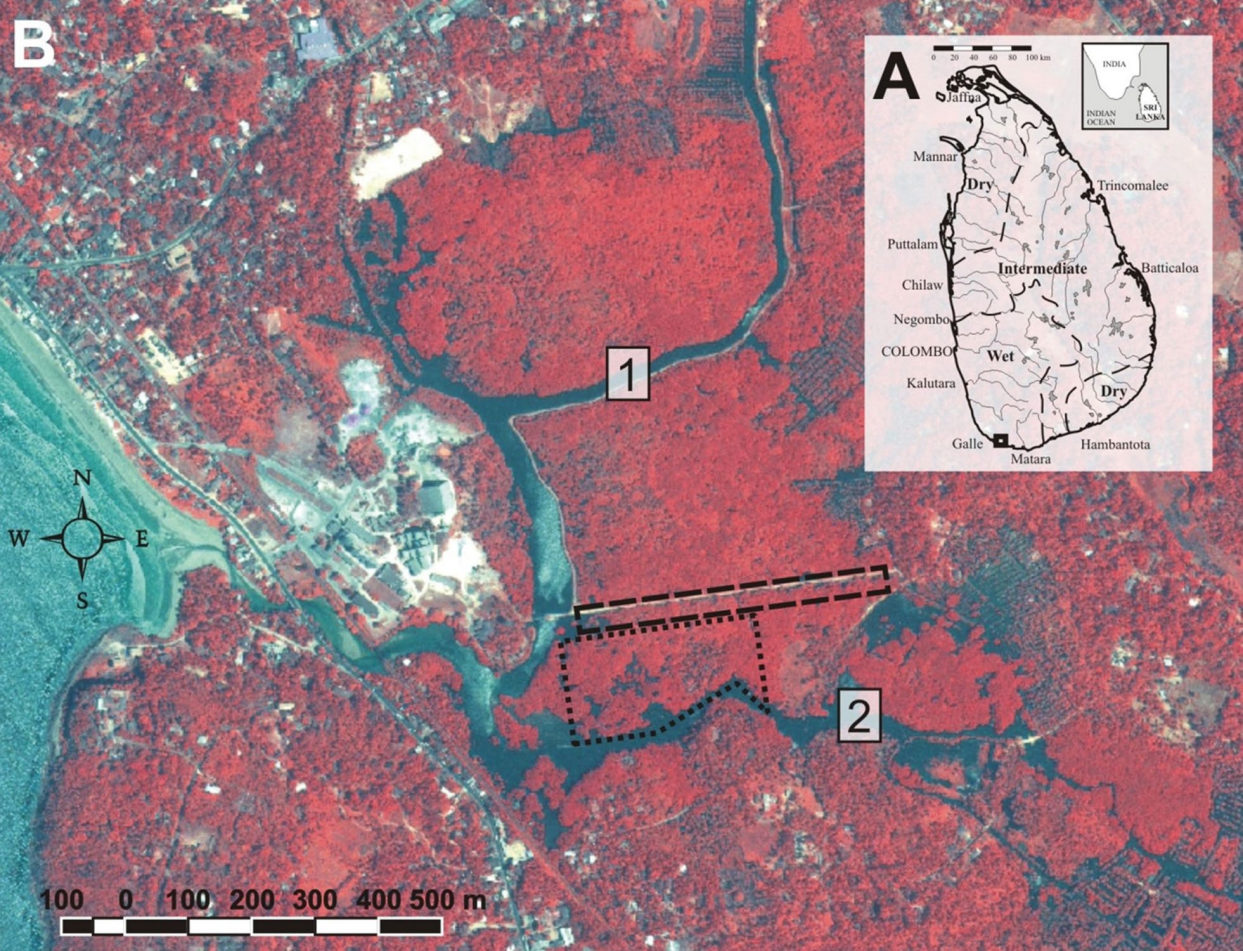
A Map of Sri Lanka, indicating the major cities, rivers and climatic zones (adapted from [44]). The small black box represents the area in B. B Satellite image of the study area (2004), indicating sampling sites (adapted from [47]). The dashed black box represents the littoral fringe adjacent to the raised motorable track, whereas the dotted black box represents the mangrove forest interior. Numbers 1 and 2 indicate, respectively Galu Ganga and Thalpe Ela

All observations, records and experiments were carried out at a mangrove forest located in southern Sri Lanka, between Galle and Unawatuna (06°01′N, 80°14′E). This mangrove, situated in the wet climate zone of Sri Lanka (Fig. 1), is located at about 600 m from the Indian Ocean. It covers a surface of 1.5 km^2^ and two rivers run through the mangrove forest: the Thalpe Ela, discharging into the ocean, and the Galu Ganga, a tributary of the former.

The dominant tree species composing the forest are *Bruguiera gymnorrhiza*, *B. sexangula*, *Excoecaria agallocha*, *Heritera littoralis* and *Rhizophora apiculata* [43, 44]. The vegetation structure of this forest changed various times since the 1950s due to major human activities carried out both within and around the mangrove [43, 45–47] and to the indirect effect on the potential predation on propagules by crabs [32]. One of the most impacting infrastructures was built in 1982, when the Galu Ganga was deepened and broadened: the sediment of the river was used to construct an earthen road that follows the river and continues through the mangrove forest, at an average height of 50 cm above the mean sea level (Fig. 1).

At the southern Sri Lanka coast, the spring tidal amplitude is less than 1 m [48] and can change as little as 15 cm per week throughout the year in the studied area [43]. Thus, a true tree zonation guided by the tide is virtually absent. However, the forest is an uneven mosaic of shallow ponds and small emerged patches of sediment, primarily resulting from the burrowing activities of the mangrove mud lobsters (*Thalassina anomala*), which are often occupied by the more terrestrial species *Excoecaria agallocha* and the mangrove associate *Acrostichum aureum* on the top, and *Rhizophora apiculata* at the margin [49].

The macrobenthic community colonising the mangrove is mostly represented by sesarmid crabs and the potamidid gastropod *Terebralia palustris* [32]. Although the relative frequency of crab species can change in the distinct parts of the forest, the most common crab species are *Neosarmatium smithi*, *N. malabaricum*, *N. asiaticum* (formerly *N. meinerti*) [50, 51], *Muradium tetragonum*, *Perisesarma dusumieri*, *Parasesarma bengalense* and *P. plicatum*. Crab species of the genera *Neosarmatium* and *Muradium* feed on fallen leaves, collect them and store litter into their burrows, enhancing the carbon sink function of mangrove forests soils [31].

Within the studied mangrove area, we selected two areas under different inundation regimes and with diverse granulometry of the soil. The first area was located within the natural mangrove area (henceforth referred to as the natural forest floor, FF), while the second one was a new portion of the forest, near the road bed, colonised by the mangrove flora and fauna over the last 20 years (henceforth referred to as the elevated man-made littoral fringe, ELF). The ELF differs from the FF for the granulometry of the soil, since it was built using the sediment from the river. Moreover, and in terms of inundation time, the ELF is seldom and unpredictably covered by water mainly during rainy seasons [32].

### Characterisation of abiotic factors and biotic assemblages at the two experimental sites

Field sampling and observations were performed during the wet season (July to August) when the crab activity is more relevant. Twenty 2 m × 2 m plots were randomly selected in both sites and the trees present recorded.

The presence and relative abundance of crab species of the genera *Neosarmatium* and *Muradium* were assessed using the field techniques described by Skov et al. [52] for large burrowing sesarmid species. All crab burrows present in the plots were recorded and attributed to the different species. Burrows of *M. tetragonum* were easy to distinguish from the *Neosarmatium* ones: the latter start with funnels almost vertical to the soil surface, whereas the former had the first part of the burrows built at very narrow angles to the soil surface. The burrows of *N. malabaricum* were easily attributed using the diameter of the aperture of the entrance. The average carapace width of *N. malabaricum* is smaller (CW ± SD = 23 ± 1 mm) than either *N. asiaticum* (CW 36 ± 4.2 mm) or *N. smithi* (CW 36 ± 3.1 mm), thus the size of the entrance could identify the smaller species. However, for both the smaller and the larger *Neosarmatium* burrows, their number was crosschecked with visually counted crabs, at their peaks of activity, during various days to identify the residents for each burrow [52].

As environmental factors, we recorded tree canopy cover, soil granulometry, surface salinity and temperature, since these factors have been reported to affect crab assemblages [7]. A digital camera was placed on the ground at the centre of each plot and a picture of the canopy cover was taken. In the picture, the extent of the plot was delimited using poles placed at its corners and visible in the picture. Each picture was then analysed using the software ImageJ, to assess the total tree cover and percentage contribution of each tree species. Soil cores (5 cm wide, 20 cm deep) were collected from a subsample of 5 plots in each site and analysed for grain size distribution and organic content following standard procedures [53]. For each core, a sample of 100 g wet weight was dried and sieved following standard protocols, and median particle diameter, quartile deviation and skewness were calculated. For organic content, a sample of 30–50 g wet weight was dried, weighed, and then incinerated in a muffle furnace at 500° C for 24 h and reweighed. The salinity of pore water and of permanent pools adjacent to the 20 plots was assessed using a salinity refractometer (Atago). The temperature of the soil surface was measured in a subsample of five randomly chosen plots during two 24 h cycles.

### Feeding experiments and behavioural observations on crab species

Feeding experiments and behavioural observations on intra- and inter-specific competitive interactions were carried out in 20 randomly selected 4 m^2^ plots at both the ELF (a stretch of about 30,000 m^2^ near the man-made road, Fig. 1) and FF (an area of about 70,000 m^2^ inside the forest, Fig. 1) sites. Before starting an experimental session, all existing fallen leaves were carefully removed to standardise food availability and quality in each experimental plot. Then, ten green fresh leaves, i.e. directly collected from the branches of each of the three most abundant mangrove trees (i.e. *Bruguiera* spp., *R. apiculata* and *E. agallocha*) were randomly put within each plot and their fate was followed for one hour. During each observation session, two different observers, placed up on the trees or behind the *Thalassina anomala* mounds not to disturb the crabs, recorded the following data: (1) the time at which each leaf was collected by a crab, (2) the crab species collecting the leaf, (3) every intra- and interspecific encounter and conflict, (4) the species of the crabs involved in this encounter, (5) the final winner, and (6) the total number of crabs observed. For 15 days, these experiments were repeated five times per day at different times of the day (day, night and twilight) for a total of 75 observation sessions per site. At both night and twilight, the observations were carried out with the help of red beam flashlights, in order to not disturb the active animals [54].

### Statistical analysis

The differences in grain size composition between the two intertidal belts were tested by mean of two-way permutational analysis of variance (PERMANOVA) [55], with intertidal belt and grain size categories as orthogonal and fixed factors. A set of three independent estimates of weights of single grain category from various plots was utilised.

Differences in total tree cover, total crab densities and densities of each species between the two belts were tested by univariate and multivariate PERMANOVA one-factor designs. In all cases, similarity matrixes were computed using Bray–Curtis distance on forth-root transformed data, since Levene test revealed heteroscedasticity of data. The contribution of the various species of trees and crabs to the differences found between belts was assessed using the SIMPER test.

A univariate PERMANOVA two-factor full factorial design with experimental site (fixed and orthogonal) and time of the day (fixed and orthogonal), was used to test differences in feeding activity, i.e. number of crabs recorded to collect leaves. The observations were divided into three temporal groups: night, day, and a group comprising dawn and dusk periods (i.e. twilight). The amount of leaves collected within 1 h of observation at the different sites and in different times of the day, both factors fixed and orthogonal, was tested using a two-way univariate PERMANOVA design. To test for differences in leaves removal among species, an ANOVA three-factor design was used, with all three factors (leaf species, habitat and period of the day) fixed and orthogonal. Since the data recorded for the three different leaf species on a same plot were dependent, for every period of the day we picked up *at random* the data on a single leaf species from five plots and disregarded the data of the other two species. Then, a χ^2^ test was applied to compare the frequency distribution of the inter-specific interactions won by each species.

Multivariate analyses were performed using the PERMANOVA+ routines for PRIMER 7 and were based on 9999 permutations [56, 57], while univariate analyses (ANOVAs and χ^2^ test) were performed using GMAV 6 program (University of Sydney, Australia) and PAST v. 2.14 [58]. In the text, results are expressed as average ± SE.

## Results

### Characterisation of abiotic factors and biotic assemblages at the two experimental sites

**Fig. 2:**
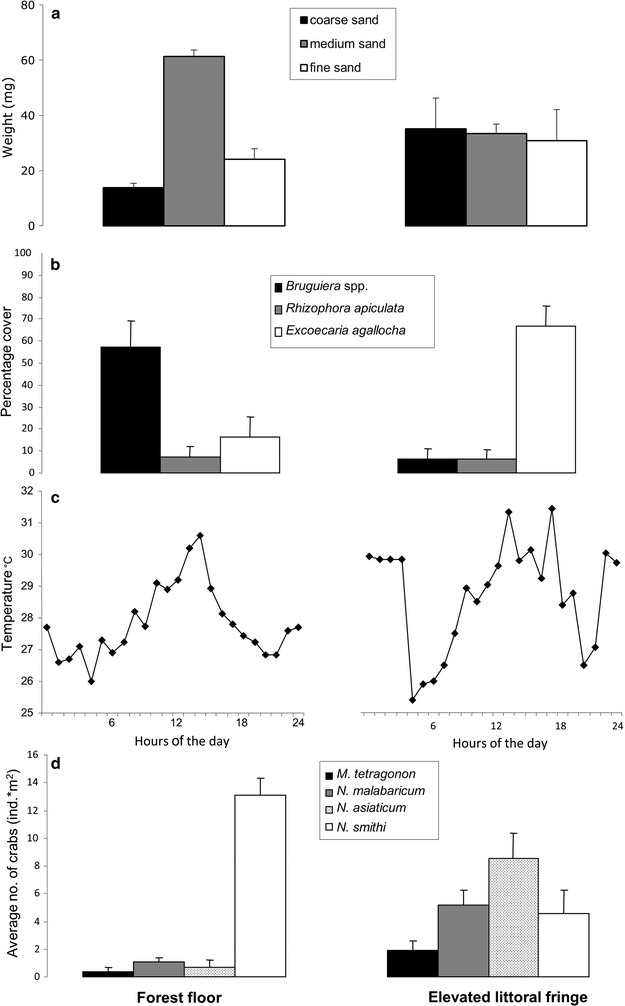
Characterisation of the two study sites. The soil texture (**a**), the percentage cover of the different trees (**b**), the pattern of surface temperature along the 24 h (**c**) and the abundance of the different crabs (**d**) are shown for the forest floor and the elevated littoral fringe, respectively

The grain size composition was significantly different between the two areas (interaction factor site × grain size category Pseudo-F = 4.77, df = 2, p = 0.03; PERMANOVA test, Fig. 2a). In particular, the soil at the FF area showed a higher amount of medium-sized sand with respect to both coarse and fine sand (t = 12.82, p < 0.001 and t = 5.35, p < 0.01, respectively, post hoc test), while the soil in the ELF was composed almost equally by very fine, medium and coarse sand (Fig. 2a). The pore water and the water contained in the permanent pools at both the sites never reached more than 1‰ of salinity, a condition typical of the wet season in this area [12].

The floral composition was different between the two sites (Pseudo-F = 33.36, df = 1, p < 0.001; PERMANOVA test, Fig. 2b). *Bruguiera* spp. dominated the FF area while it was not abundant at the ELF (average abundances 56.98 and 6.48, respectively, contribution to dissimilarity 44.26%, SIMPER test, Fig. 2b). *E. agallocha* was not abundant in the FF, but it was dominant at the ELF (average abundances 16.36 and 67.06, respectively, contribution to dissimilarity 44.21%, SIMPER test, Fig. 2b); *R. apiculata* was always the less common tree species and there was no difference between the two belts (Fig. 2b).

The total tree cover was also different between the two intertidal belts (Pseudo-F = 4.15; df = 1; p = 0.011, PERMANOVA), with the FF more shaded than the ELF (91.34% ± 1.15 and 81.88% ± 3.87, respectively). This difference in tree cover affected the temperature of the soil surface: under the forest the average surface temperature during the day was 28.88 °C ± 0.30, while in the ELF the average temperature was 29.53 °C ± 0.38 (Fig. 2c).

The total abundance of crab species was significantly higher at the ELF than in the FF (20.31 ± 1.37 and 15.30 ± 1.01, respectively, Pseudo-F = 4.97, df = 1, p = 0.025; PERMANOVA test) and the relative frequency of the diverse species was different (Pseudo-F = 53.45, df = 1, p < 0.001; PERMANOVA test) (Fig. 2d). The dominant species at the FF was *N. smithi*, being 85.9% of the recorded crabs, and it was far more common than at the ELF (average abundances 13.14 ind/m^2^ and 4.59 ind/m^2^, respectively, contribution to dissimilarity 23.71%, SIMPER test) (Fig. 2d). On the other hand, *M. tetragonum*, *N. malabaricum* and *N. asiaticum* were more common at ELF than at FF. At the ELF, *N. asiaticum* outnumbered the other species, representing up to 42.2% of the total crabs and contributing to the dissimilarity between the two by 33.45% (SIMPER test) (Fig. 2d).

### Feeding experiments and behavioural observations on crab species

**Fig. 3:**
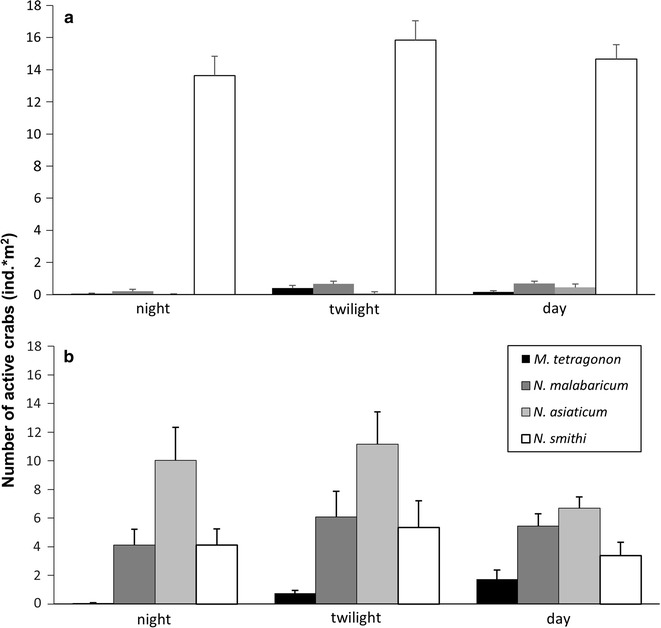
Feeding activity of the four observed crab species as recorded on the forest floor (**a**) and at the elevated littoral fringe (**b**) during nocturnal, twilight and daytime hours

The number of feeding individuals belonging to the four focal species was proportional to their densities (Fig. 3a, b; Table 1). The main litter consumer on the FF was *N. smithi*, while *N. asiaticum*, *N. malabaricum* and *M. tetragonum* were more common in feeding on the leaves from the experimental plots placed in the ELF. The feeding activity of *N. smithi*, *N. asiaticum* and *N. malabaricum* were evenly distributed along the 24 h in both the sites, showing no temporal segregation among the species activity (Table 1). On the other hand, *M. tetragonum* showed to be more active at the ELF than at the FF site during the hottest hours of the day (t = 3.85, p < 0.001, post hoc test).

**Table 1:** Results of the two-way PERMANOVAs performed on the numbers of each experimental crab species feeding at the two study sites during different times of the day

Source	DF	*M. tetragonum*			*N. malabaricum*			*N. asiaticum*			*N. smithi*		
		MS	P-F	P	MS	P-F	P	MS	P-F	P	MS	P-F	P
Site (Si)	1	4.88	17.97	< 0.001	32.64	79.39	< 0.001	61.63	96.89	< 0.001	69.24	96.96	< 0.001
Daytime (Dt)	2	0.40	1.48	0.24	0.16	0.40	0.67	0.33	0.52	0.59	0.12	0.17	0.85
Si × Dt	2	1.09	4.03	0.02	0.92	2.23	0.11	0.70	1.09	0.25	0.36	0.36	0.70
RES	84	0.27			0.41			0.64			0.71		
TOT	89				89			89			89		

For each test and each factor (*RES* residuals), *DF* the degrees of freedom, *MS* mean square, *P-F* values of Pseudo-F statistic, *P* the probability level, are shown

**Fig. 4:**
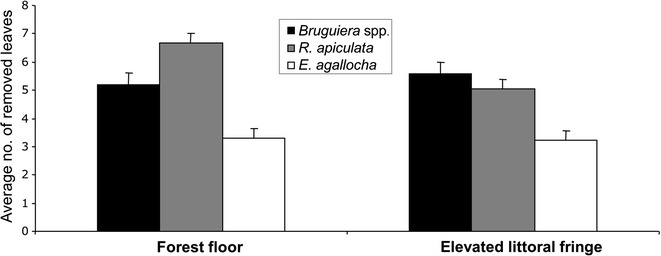
Number of leaves of the different species collected in the experimental plots

During our experiments, the total number of leaves collected by the experimental crabs after 1 hour of observation did not vary between the two sites (Pseudo-F = 1.35, df = 1, p = 0.25; PERMANOVA test, Fig. 4) at different times of the day (Pseudo-F = 0.39, df = 2, p = 0.68; PERMANOVA test, Fig. 4). Overall, there was a strong difference in leaf removal among species, with the leaves of *R. apiculata* and *Bruguiera* spp. significantly more frequently removed over the ones of *E. agallocha* (SNK test, Table 2, Fig. 4). The removal of the Rhizophoraceae was constant over the day and did not change between the two sites (Table 2).

**Table 2:** Results of the 3-factor ANOVA performed on leaves removal at the two experimental sites, within the different daytimes

Source	DF	MS	F	P
Leaves (Le)	2	2.15	21.17	< 0.001
Site (Si)	1	0.18	1.77	0.19
Daytime (Dt)	2	0.01	0.05	0.95
Le × Si	2	0.13	1.27	0.29
Le × Dt	4	0.07	0.7	0.60
Si × Dt	2	0.20	1.93	0.15
Le × Si × Dt	4	0.17	1.66	0.17
RES	72	0.10		
TOT	89			

*DF* degrees of freedom, MS mean square, *F* values of F statistic, *P* the probability level, are shown

A total of 1671 leaves was collected by the crabs during the 15 days of observations and successfully stored in their burrows. We also observed a total of 121 intra- and inter-specific agonistic interactions in which a leaf, previously collected by one crab, was stolen by another crab, which successfully stored it in its burrow. In the two habitats, these acts of agonistic competition had different characteristics, both in terms of species involved and number of interactions (Table 3). At the ELF (i.e. the densest site) we recorded a higher number of agonistic interactions than on the FF (82 and 39, respectively: Table 3). Moreover, at the ELF we observed fights involving all pairs of species present in the area, while on the FF intra-specific interactions were infrequent and some species pairs never interacted (Table 3). The number of leaves won from an opponent was proportional to the total number of leaves collected and stored in the burrows for each species at both intertidal levels (χ^2^ = 3.59, df = 3, p = 0.30 and χ^2^ = 2.02, df = 3, p = 0.56), showing that the number of agonistic interactions was related to the frequencies of feeding activity. However, no species was able to steal a single leaf in 29 agonistic encounters involving as an opponent *N. smithi*, regardless of the intertidal area. At the ELF, *N. asiaticum* was the most successful in conquering a leaf in an aggressive contest, but it was never able to conquest a leaf from *N. smithi* (Table 3). The same was true for *M. tetragonum* and *N. malabaricum* that were often engaged in interactions for the leaves but never able to succeed when the opponent was a *N. smithi*. This latter species was the only one capable of winning agonistic interactions observed at FF site (Table 3).

**Table 3:** Feeding experiments and intra- and inter-specific agonistic interactions observed at the two intertidal levels

Elevated littoral fringe						
Feeder	TOT removed	Stolen	*M. tetragonon*	*N. malabaricum*	*N. asiaticum*	*N. smithi*
*M. tetragonon*	50 (5.85%)	4	1 (25.0%)	0	3 (75.0%)	0
*N. malabaricum*	222 (26.03%)	14	1 (7.1%)	11 (78.6%)	2 (14.3%)	0
*N. asiaticum*	422 (49.47%)	46	6 (13%)	18 (39.1%)	22 (47.8)	0
*N. smithi*	159 (18.64%)	18	1 (5.6%)	7 (38.9%)	8 (44.4%)	2 (11.1%)

Forest floor						
Feeder	TOT removed	Stolen	*M. tetragonon*	*N. malabaricum*	*N. asiaticum*	*N. smithi*
*M. tetragonon*	10 (1.17%)	0	0	0	0	0
*N. malabaricum*	28 (3.28%)	0	0	0	0	0
*N. asiaticum*	14 (1.64%)	1	0	0	1 (100%)	0
*N. smithi*	766 (89.80%)	38	5 (13.2%)	6 (15.8%)	0	27 (71.0%)

For each of the observed species (feeder), the total numbers of leaves removed (TOT removed), the numbers of leaves stolen from another crab in agonistic encounters (Stolen) and numbers (and percentages) of agonistic interactions for each pair of species are shown. 0 means no interactions between pairs of species

## Discussion

Our data showed how the direct anthropogenic influence on this Sri Lankan mangrove forest resulted in a true ecological modification of the area, with a clearly defined original forest, colonising the eulittoral zone, and a newly formed littoral fringe, which was promptly re-colonised by mangroves and crabs. We took advantage of this situation in planning this study, since tree and crab community composition and density in the new littoral fringe proved to be different from the lower eulittoral forest area. The elevated littoral fringe was a harsher environment respect to the mangrove forest floor due to almost no inundation periods, high surface temperature and by the dominance of *E. agallocha*, known to be a terrestrial mangrove associate [59]. However, in terms of crab assemblage, this habitat has been colonised by a denser and more diverse assemblage of litter-feeding sesarmid crabs than the forest floor. The smaller litter feeders (*M. tetragonum* and *N. malabaricum*) were definitely most abundant here, while the rest of the forest was strongly dominated by *N. smithi*.

The higher crab density recorded in the littoral fringe as opposed to the forest floor is rather unexpected, since this habitat has significantly lower tree cover than the forest and, consequently it provides lower amounts of litter, presumably resulting in a shortage of food for these sesarmids. Moreover, the elevated littoral fringe was dominated by *E. agallocha*, which produces litter rich in a lattice poisonous for humans [59] and was by far the less eaten food among all the tested crab species. Although sesarmid crabs can rely on food sources other than leaf litter [22, 60–62], this is still the major component of their diet [see 21, 63–66]. Indeed, both shortage of litter and a diet based on litter of poor quality proved to have negative consequences on sesarmid crabs, as proved for *Perisesarma messa* [67] and for *Parasesarma affine* and *Perisesarma bidens* [68].

Thus, why we found a denser and more diverse assemblage of sesarmids at the man-made elevated littoral fringe than on the natural forest floor? And why were all the species except *N. smithi* more abundant in this unfavourable habitat? A reasonable explanation comes from the strong interference competition recorded during our feeding experiments with a noticeable highest aggressiveness and dominance showed by *N. smithi* towards the other litter-feeding species.

Inter- and intra-specific interference competition events appeared to be very frequent at both intertidal levels and throughout the 24 h. All the study species showed to feed mainly on the same food, i.e. Rhizophoraceae leaves, and this unquestionably enhanced the direct competition for food. All species were also actively feeding at any time of the day, showing no temporal segregation, despite previous data from East Africa reported a preference for diurnal activity by *N. smithi* [69]. Virtually interference competition is almost always asymmetric, with some individuals/species dominating the others [70]. In our case, *N. smithi* individuals were always the dominant competitors at both areas, never being defeated in any inter-specific interactions. The dominance of *N. smithi* in gathering mangrove litter may prevent other species to colonise the forest floor and force them to move in a new habitat although less favourable. We, thus, suggest that the lower limit of zonation for *N. asiaticum*, *N. malabaricum* and *M. tetragonum* within our study area was primarily set by the competition for food with *N. smithi*.

Dominance is frequently related to the size and force of the opponents [21, 70–73]. Actually, *N. smithi* was not the largest of the species observed at our study site, since it is very similar in dimensions to *N. asiaticum,* and it is not endowed with any special “weapon” (i.e. the largest chelae: [71, 72]). Since during our observations we never observed an agonistic encounter towards *N. smithi*, we propose that the dominance of *N. smithi* can be due to its speed and readiness to exit its burrow, instead of its effective force (as already shown for other herbivorous mangrove crabs: [21]).

In intertidal ecology, it has been often reported that the lower limit of a species’ distribution is determined by biotic factors, such as predation pressure and competition for limiting resources [74–76], while the upper limit is set by abiotic factors, such as temperature, salinity, and water supply [74–76]. Moreover, it is known that higher intertidal fringes expose animals to harsher abiotic conditions than lower intertidal areas [75], thus hosting, under competitive conditions, mainly inferior competitors [36, 77–79]. The present study is in line with the above results and indicates, for the first time in mangrove ecosystems, that interference competition for feeding resources may be a major force shaping the spatial distribution of sesarmids, setting their lower distributional limit. This biotic factor likely acts in synergy with abiotic factors, such as salinity, soil temperature and inundation time, that vary between the two experimental habitats and over the dry and wet seasons in Sri Lanka [80]. At the same time, we explain the low density of *N. smithi* in the less favourable elevated littoral fringe, hypothesising that its upper limit of distribution can be set by physical factors (elevated temperature, low humidity of the soil and high exposure to the sun light). Although nothing is known about the biology and physiology of *N. asiaticum*, *M. tetragonum* and *N. malabaricum*, the seaward distribution of *N. smithi* all along the East African coast [81] and lower tolerance to changes in salinity recorded for *N. smithi* with respect to its east African congeneric *N. africanum* (old name *N. meinerti* [51]) [82] may suggest that *N. smithi* could be more vulnerable to harsh environments than other congeneric species and, more generally, than other sesarmids.

## Conclusions

The historical hypotheses about the drivers of spatial distribution of sesarmids mostly imply a strong linkage between the litter-feeding species and their preferred trees [6, 7]. Those hypotheses are barely corroborated by the vast literature on the subject, which shows how these herbivorous crabs exerted no or very weak preferences for different mangrove leaves [63, 65, 67]. Our data, in fact, cannot be explained by such theories, since all experimental crabs living in the *E. agallocha*-dominant littoral fringe avoided its leaves and may be thus facing food shortage.

The role of interference competition in shaping zonation and spatial distribution of mangrove crabs has been underestimated in mangrove ecology, and therefore our study is novel in enlightening a strong effect of this biological factor in structuring mangrove macrobenthic assemblages. However, we are also aware that our findings need to be corroborate by other field observations and trials. In particular, we recognise the importance of performing manipulative removal or exclusion experiments as historically done in other intertidal habitats [83], despite such an approach is very difficult or even impossible to be applied in mangroves with large sesarmid crabs [84, 85]. Finally, this study adds interference competition for food as a key factor in the complex interplay of biotic (predation, competition for space) and abiotic environmental factors determining the spatial and distribution patterns of mangrove fauna.
